# Evaluation of Mucoadhesive Nano-Bilosomal In Situ Gels Containing Anti-Psychotic Clozapine for Treatment of Schizophrenia: In Vitro and In Vivo Studies

**DOI:** 10.3390/ph17101404

**Published:** 2024-10-21

**Authors:** Marwa H. Abdallah, Mona M. Shahien, Hemat El-Sayed El-Horany, Enas Haridy Ahmed, Hanan M. El-Nahas, Nourhan A. Abdulla, Tarek M. Ibrahim

**Affiliations:** 1 Department of Pharmaceutics, College of Pharmacy, University of Ha’il, Ha’il 81442, Saudi Arabia; 2 Department of Pediatrics, College of Medicine, University of Ha’il, Ha’il 81442, Saudi Arabia; m.shahin@uoh.edu.sa; 3 Department of Biochemistry, College of Medicine, University of Ha’il, Ha’il 81442, Saudi Arabia; h.elhorany@uoh.edu.sa; 4 Department of Medical Biochemistry, Faculty of Medicine, Tanta University, Tanta 31511, Egypt; 5 Department of Anatomy, College of Medicine, University of Ha’il, Ha’il 81442, Saudi Arabia; e.haridy@uoh.edu.sa; 6 Department of Anatomy and Embryology, Faculty of Medicine, Ain Shams University, Cairo 11566, Egypt; 7 Department of Pharmaceutics, Faculty of Pharmacy, Zagazig University, Zagazig 44519, Egypt; hananelnahas@gmail.com (H.M.E.-N.); nourhan.adel.moh94@gmail.com (N.A.A.); tarekmetwally333@gmail.com (T.M.I.)

**Keywords:** clozapine, bilosomes, definitive screening design, in situ gel, ELISA, schizophrenia

## Abstract

**Background/Objectives:** Patients with schizophrenia have significant challenges in adhering to and complying with oral medicines, resulting in adverse consequences such as symptom worsening and psychotic relapse. **Methods:** This study aimed to develop clove oil-based bilosomes using definitive screening design (DSD) to maximize the anti-schizophrenic action of clozapine and promote its nose-to-brain delivery. The target was to optimize the physicochemical properties of bilosomes and incorporate them into mucoadhesive intranasal in situ gels, searching for augmented ex vivo and in vivo clozapine delivery. **Results:** The bilosomes’ particle size was decreased by increasing the span, SDC, and clove oil amounts. In addition to using a high lipid amount, the aforementioned components also helped increase the entrapment efficiency values. Increased zeta potential was only observed by increasing surfactant amount and reducing clozapine concentration. After incorporation of optimized liquid clove oil-based bilosomes, which had a spherical nano-sized vesicular shape, into P 407-dependent gels, an HPMC (2% *w*/*w*)/P 407 (20% *w*/*w*)-containing formulation (G6) was selected as an optimized gel owing to its acceptable gelation time (13.28 s), gel strength (27.72 s), viscosity (12,766.67 cP), and mucoadhesive strength (4273.93 dyne/cm^2^). The optimized G6 exhibited higher J_ss_ (50.86 μg/cm^2^·h^−1^) through the nasal mucosa compared to the control gel (23.03 μg/cm^2^·h^−1^). Compared to the control gel, G6 displayed higher relative bioavailability (491.37%) than a commercial tablet (264.46%). Following ELISA analysis, dopamine and serotonin were significantly reduced, while BDNF was remarkably increased after administration of optimized G6 into schizophrenic rats. **Conclusion:** Our study indicates the potential of intranasal bilosomal gels in upgrading the anti-schizophrenic and neuroprotective activity of clozapine.

## 1. Introduction

Schizophrenia, a psychiatric condition, is a severe chronic mental illness marked by significant aberrations in cognition, perception, emotions, behavior, and self-identity. It encompasses prevalent psychotic phenomena such as paranoia, auditory hallucinations, and delusions as positive symptoms. The management of schizophrenia has involved two primary groups of anti-psychotics: first-generation (typical) medications, such as chlorpromazine, haloperidol, and flupenthixol; and second-generation (atypical) medications, such as clozapine, risperidone, aripiprazole, and olanzapine. Second-generation medications are more effective in decreasing relapse rates and addressing both the positive and negative symptoms of schizophrenia compared to traditional medications that just target positive symptoms [1].

Clozapine is one of the atypical anti-psychotic drugs for resistant schizophrenia [2]. Compared to conventional anti-psychotics, clozapine is superior in alleviating the positive and negative symptoms in individuals with severe schizophrenia [3]. The oral administration of clozapine is associated with a number of common adverse effects, including those affecting metabolism, the gastrointestinal tract, the heart, and blood [4,5]. Clozapine is classified as a class II medication in the Biopharmaceutical Classification System. This classification indicates that it has a high permeability and a low solubility. It has a high rate of first-pass metabolism and a poor bioavailability (>27%) after oral administration, although it is absorbed rapidly and completely [6]. Moreover, half of the individuals with schizophrenia do not comply with therapy and do not take oral medications, which may lead to unsatisfactory therapeutic results. In recent years, various clozapine formulations have been created for injectable administration, such as polymeric nanocarriers [7], proniosomes for transdermal administration [8], and nanoparticles [9] to decrease disturbance and enhance compliance in schizophrenic patients. Unfortunately, there are a few drawbacks to these formulations; for example, there is a possibility of particle aggregation or sedimentation, where the drug loading is restricted and there is a risk of storage leakage [10]. Furthermore, several studies have documented the effective delivery of clozapine via the intranasal route with various techniques. Velupula and Janapareddi (2020) improved the ex vivo flow rate by formulating clozapine as a mucoadhesive microemulsion compared to a drug solution [11]. Tan et al. (2022) reported that the intranasal nanoemulsion gel of clozapine has the ability to improve the poor pharmacokinetic properties of clozapine [12]. Furthermore, Sayed et al. (2021) examined the efficacy of clozapine targeting to the brain using Technetium-99 m-radio-tagged mixed micelles [13].

Recent studies have shown that the intranasal route can increase bioavailability and serve as an alternative to oral delivery. Due to its wide surface area, high blood supply, and porous endothelium membrane, the nasal mucosa allows for greater drug absorption and bypasses first-pass metabolism. Furthermore, it is able to pass the blood–brain barrier, which eliminates the requirement for systemic administration and the risks associated with it [13].

**Figure 1:**
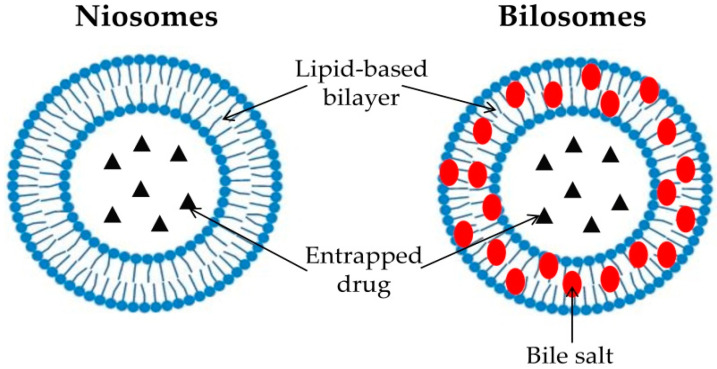
Schematic comparison between niosomes and bilosomes.

Classic liposomes and niosomes are noted to have challenges, including inadequate trapping, low solubility, and restricted tissue permeability, resulting in insufficient drug penetration into deeper skin layers with negligible systemic absorption. From this viewpoint, the modification of these vesicular nano-carriers to generate novel platforms can enhance drug diffusion through the nasal membrane. Bilosomes are modern ultra-deformable nano-vesicle systems that resemble niosomes in composition but are enhanced with bile salts in addition to the presence of non-ionic surfactants (Figure 1). Bile salts function by reducing the lipid bilayers’ surface tension in bilosomes. Moreover, they can enhance the flexibility and deformability of bilosomes, enabling them to traverse the nasal mucosa and thus promoting the drug deep penetration [14]. Bile salts are identified as a membrane softening agent and they exhibit electrostatic stabilization properties, resulting in stable bilosomes. Additionally, they have fluidizing properties that can enhance drug delivery through biological membranes [15]. El-Nabarawi et al. [16] indicated that the efficacy of bile salts in boosting penetration and absorption is attributed to their ability to augment the solubility of water insoluble medicines and increase the fluidization of membranes. Nair et al. [17] indicated that bile salts are beneficial due to their non-toxicity, safety, and biological compatibility.

On the other side, the presence of lipid constituents can fluidize the skin layers, making them more permeable to drugs; thus, a systemic uptake of drugs can be maximized [18]. Non-ionic surfactants, such as span 60, are the primary bases for the preparation of bilosomes. They facilitate the entrapment of greater amounts of a loaded drug inside bilosomal vesicles and increase their permeation through biological surfaces [19]. In addition, they can boost the stability of bilosomes by producing an interface with negative charges owing to their hydroxyl groups that are adsorbed on the vesicles’ surfaces [20]. Cholesterol can be also used for increasing the stability of vesicle membranes and improving the encapsulation efficiency of vesicles [21]. In our study, clove oil was used as an additional penetration enhancer during the bilosomes’ preparation. Clove oil has been reported to have a valuable absorption profile that could help with drug partitioning into biological surfaces. The presence of terpenes as a main component of clove oil gives it a beneficial penetration-enhancing action [22].

The durability and deposition of nano-vesicular systems in the mucosal membrane could be enhanced through integrating them into polymeric in situ gels [23]. To optimize the efficacy of the formulated intranasal bilosomes, it is essential to prolong the nasal mucociliary transit time, therefore enhancing the retention of the administered dosage form inside the nasal cavity and promoting drug absorption. The generated bilosomes were included into a mucoadhesive in situ gelling system with acceptable characteristics that could withstand fast mucociliary clearance.

Response surface methodology (RSM), a collection of mathematical and statistical techniques, may be employed to effectively optimize various formulations. The RSM approach can identify the amounts of various independent variables that provide favorable dependent responses and affect the quality of process outputs [24]. This study employed the definitive screening design (DSD) as an effective instrument inside the RSM. DSD is a novel category of three-level design that is utilized for the definitive screening of qualitative and quantitative variables. DSD has different useful features from other conventional RSM and factorial designs, notably where the number of runs necessary for DSD creation is one greater than twice the number of variables [25]. It depends on minimizing the number of experiment runs and is suitable for systems that require studying a large number of factors, thus providing low cost and time [26].

To the authors’ knowledge, no prior research has been published on the incorporation of clozapine into bilosomal in situ gel systems, nor the investigation of its anti-schizophrenia efficacy following intranasal delivery. The objective of this work was to examine the impact of several components at varied concentrations on the features of bilosomes, including entrapment efficiency, particle size, zeta potential, and cumulative in vitro drug release, utilizing the DSD. The optimum formulation was incorporated into an in situ gel, and assessments of bilosomal in situ gels were performed. Ex vivo skin permeation investigation was conducted on the optimized bilosomal in situ gel formulation to control the performance of in situ gel in terms of their safety and capacity to have the clozapine penetrate into the mucosal membrane. Furthermore, the optimal intranasal in situ gel was examined for its ability to augment the anti-schizophrenic and neuroprotective efficacy of clozapine.

## 2. Results and Discussion

### 2.1. Analysis of DSD

#### 2.1.1. Influence of Independent Factors on Particle Size

The polynomial equation showing the impact of tested factors on the particle size of bilosomes is given below:

(1) Particle size (nm)=+351.71+53.19 A−28.67B−56.87C−26.80 D+37.99 E.

When the sign of the factor’s coefficient was positive, it meant that increasing the level of the specific factor can increase the response. This is termed as a positive effect. In contrast, when the sign of the factor’s coefficient is negative, it means that increasing the factor level can decrease the response. This is termed as a negative effect. The previous equation demonstrated that the PL amount (A) and drug amount (E) showed positive effects on the particle size, while the span amount (B), SDC amount (C), and clove oil (D) showed negative effects. In addition, the type of span (F) showed no significant effect on the vesicles’ size (Figure S1 and Table 1).

Measurement of the particle size of bilosomes is crucial because small particle size values can influence the encapsulation of drugs and stability of vesicles. This will help bilosomes to penetrate the biological membranes easily, achieving promoted drug permeation and enhanced in vivo outcomes [27]. As summarized in Table 1, the mean particle size of different bilosomal vesicles fluctuated from 215.50 ± 23.06 nm to 535.00 ± 22.35 nm and was affected by various formulation variables.

As illustrated in Figure S1a, there was a positive relationship between the phospholipid amount and particle size. The mean particle size of the prepared vesicles with high PL was significantly larger than those prepared with a low PL amount. One possible explanation for the large particle size was that the produced vesicles were rigid, and the increase in PL quantities led to the creation of thick structures [28].

The second variable, the surfactant amount (B), demonstrated a negative impact on the particle size (Figure S1b). Increasing the amount of surfactant led to a decrease in the particle size due to the reduction in interfacial tension between the lipid and aqueous phases. A comparable observation was previously reported while preparing the bilosomes loaded with quercetin [29] and dapsone [16].

The third variable was the amount of SDC (C), which demonstrated an adverse impact on the particle size of bilosomes loaded with clozapine (Figure S1c). Increasing the concentration of SDC resulted in decreasing the particle size. This might be attributable to the surfactant properties of bile salt (SDC), which aid in diminishing the interfacial tension and augmenting the stability of bilosomes. These results were confirmed by the previously stated literature [20,30].

It was noticed that increasing the amount of clove oil (D) led to a decrease in the bilosomes’ size (Figure S1d). This might be due to the reduced surface free energy resulting from increasing the total lipid. Our results were consistent with those documented by Kammoun et al. [31].

Our research indicates that clozapine concentration significantly influences the particle size. It was noted that an increase in drug concentration correlates with an increase in particle size (Figure S1e). Increased drug concentration could result in a greater number of drug molecules being integrated into the hydrophobic regions of the vesicles, hence increasing the space between the vesicular bilayers [32].

#### 2.1.2. Influence of the Independent Factors on Entrapment Efficiency

The polynomial equation representing the influence of the independent factors on the entrapment efficiency of bilosomes is shown below:

(2) Entrapment effeciency (%)=+56.98+14.62 A+13.19 B+11.13 C+13.79 D−6.90 E−3.96 F.

According to Equation (4), the PL amount (A), surfactant amount (B), SDC amount (C), and clove oil (D) showed beneficial effects on the entrapment efficiency, whereas drug amount (E) and the type of span (F) indicated negative impacts (Figure S2 and Table 1).

The data in Table 1 indicate that the entrapment efficiency values ranged from 24.99 ± 0.81% to 88.57 ± 1.19%.

The amount of PL considerably influenced the entrapment efficiency of the bilosomal vesicles. An increase in the entrapment efficiency was demonstrated with a corresponding increase in PL content (Figure S2a). The increase in entrapment efficiency corresponding to elevated PL amount could be attributed to the lipophilicity of clozapine as the lipophilic drug would discover an appropriate environment for encapsulation inside the lipoid phase of the vesicles [28]. Additionally, this might be ascribed to the elevated viscosity and stiffness of the membrane at increased PL amounts, resulting in decreased drug leakage from the bilosomal membranes [31].

Moreover, an increase in the entrapment efficiency was obtained with increased surfactant amount (Figure S2b). Increasing the amount of surfactant enhanced the encapsulation efficiency of the clozapine due to its role in enhancing the stabilization of the lipid membrane and decreasing the interfacial tension, which led to increased vesicle surface areas [33].

Figure S2c illustrates the effect of varying the bile salt (SDC) amount on the clozapine entrapment, which indicated that entrapment might be significantly enhanced by increasing the independent variable C. The surface-active characteristics and ionic nature of SDC might augment the repulsion between bilosomal vesicles, hence facilitating improved drug entrapment, as noted by Zewail et al. [34]. Furthermore, bile salts could enhance the flexibility of lipid bilayers upon incorporation into vesicles, leading to better drug entrapment. Consistently, it has been found that the solubility of clozapine in vesicle membranes could be augmented due to the surface-active properties of bile salts, hence enhancing its encapsulation inside bilosomes, as reported by Ibrahim [20].

Furthermore, increasing clove oil content resulted in a marked increase in the entrapment efficiency (Figure S2d). The elevation in clove oil content might have enhanced the solubility of clozapine within the vesicle’s shell, resulting in the encapsulation of a greater quantity of clozapine [33].

The concentration of clozapine significantly influenced the obtained entrapment efficiency values (Figure S2e). Bilosomal formulations created with an elevated concentration of clozapine exhibited decreased entrapment efficiency values. This might be due to the high concentration gradient that could facilitate the leakage of drugs away from the bilosomal vesicles [35].

Span 60-based vesicles exhibited a higher encapsulation efficiency compared to span 80-based vesicles (Figure S2f). These findings align with the studies conducted by Ammar et al. [36], who examined the influence of span type on the drug encapsulation in bilosomes. The alkyl chain of span 80 had an unsaturated bond that might reduce the entrapment efficiency via enhancing the vesicle permeability. The lower transition temperature (Tc) of span 80 (−12 °C) compared to span 60 (53 °C) might account for the reduced entrapment efficiency values of span 80-based vesicles [37].

#### 2.1.3. Influence of Independent Factors on Zeta Potential

The polynomial equation representing the influence of the independent variable on zeta potential is shown below:

(3) Zeta potential (mV)=+35.12+4.89 B−7.33 E.

The ANOVA analysis indicated that the surfactant amount (B) and drug amount (E) significantly influenced the zeta potential, while other variables had non-significant impacts on the zeta potential (Figure S3 and Table 1).

Zeta potential refers to the amount of the charge on the surface of bilosome vesicles in the colloidal dispersion. The zeta potential represents the extent of repulsion among vesicles and the level of physical stability [38]. Table 1 illustrates that all formulated clozapine-loaded bilosomes exhibited negative zeta potentials, varying from −21.87 ± 1.95 mV to −49.96 ± 0.98 mV. The negative charge could be ascribed to the PL and bile salt [32].

The amount of surfactant (span) significantly influenced the obtained zeta potential values (Figure S3a). The elevation of the absolute values of the zeta potential corresponding to the increase in surfactant concentration might be attributed to the charge it conferred on the vesicles’ surfaces [39]. Furthermore, the absolute zeta potential values were elevated by augmenting the surfactant amount, which is likely attributable to the increased surface area resulting from the decrease in interfacial tension induced by span [40].

The concentration of clozapine significantly influenced the zeta potential values (Figure S3b). Bilosomal formulations generated with an elevated concentration of clozapine exhibited low zeta potential values. This might be owing to the ionizable groups (four basic nitrogen atoms) in the clozapine structure that promoted a change in the zeta potential values toward neutrality [10].

#### 2.1.4. Influence of Independent Factors on Cumulative Drug Release After 8 h

**Figure 2:**
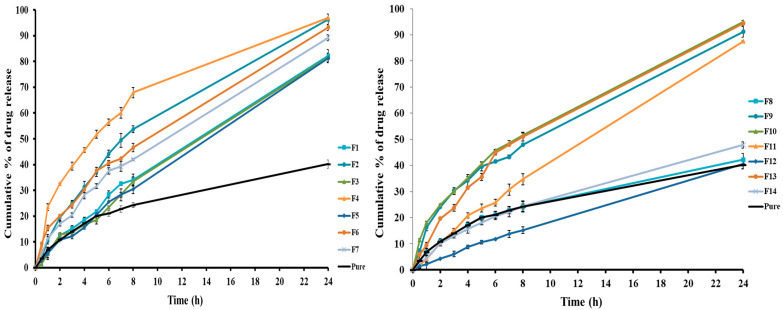
In vitro release profiles of the clozapine from clozapine suspension and clozapine-loaded bilosomes (F1–F14).

Figure 2 illustrates the in vitro release profiles, demonstrating the cumulative quantity of released clozapine from both the clozapine suspension and the generated clozapine-loaded bilosomes over time. The percentage of clozapine release at 24 h from the clozapine suspension was determined to be 40.19 ± 1.58%, and it is attributable to the inadequate solubility of clozapine [10]. The release patterns demonstrated a progressive and continuous release of clozapine from various bilosomal formulations over 24 h, and this was potentially due to the capacity of bilosomes in functioning as a drug reservoir.

The polynomial equation representing the influence of the independent variable on the cumulative percentage of drug release after 8 h is shown below:

(4) Cumulative drug release after 8 h (%)=+39.79−9.82 A+6.09 B+7.68 C+3.92 D−4.62 F.

The equation manifested that the span amount (B), SDC amount (C), and clove oil (D) showed positive effects on the drug release percentage after 8 h, while the PL amount (A) and span type (F) showed negative effects. In addition, the drug amount (E) showed an insignificant effect on the release percentage of clozapine after 8 h (Figure S4 and Table 1).

It was evident that elevating the PL amount (A) had a detrimental antagonistic impact on the percentage of cumulative drug release after 8 h (Figure S4a). This might be due to the previously disclosed observation that larger bilosomes were produced with an increase in the PL amount. Increasing the particle size often offered an increased surface area, resulting in a reduced percentage of clozapine being released [41]. Another cause might be attributed to the inclusion of a high amount of lipid in the bilosomal formulation, which enhanced the stability and contributed to the rigidification of the bilosomes’ surfaces, resulting in a reduction in the percentage of medication released in vitro [42].

Both the span amount (B) and SDC amount (C) demonstrated a substantial favorable influence on the percentage of cumulative release of the clozapine from bilosomes after 8 h (Figure S4b,c). At elevated amounts, they could enhance the flexibility of the produced vesicles, thereby augmenting the percentage of the clozapine released from bilosomal vesicles [15].

Figure S4d depicts the increment of clove oil amount that caused an increase in the release percent of clozapine from the bilosomes after 8 h. This could be due to the enhanced fluidity of the bilayers of vesicles induced by the penetration-enhancing effect of clove oil [38]. Furthermore, the high deformability of bilosomal vesicles generated by increasing the amount of clove oil would increase the drug release from the vesicles [43].

Additionally, the bilosomal systems based on span 60 exhibited an easier release and permeation of clozapine compared to those formulated with span 80 (Figure S4e). This would be due to the higher HLB value of span 60 (4.7) that enhanced the drug release than span 80 (4.3) [44]. Consistently, Ammar et al. concluded that bilosomal vesicles including span 60 showed higher in vitro permeation of ondansetron hydrochloride compared to those that contained span 80 [36].

### 2.2. Optimization of Clozapine-Loaded Bilosomal Formulation

The optimized bilosomal formulation with the highest desirability value (0.976) was chosen. Table 2 illustrates the composition of the optimized formulation, as suggested by DSD. In addition, the predicted and experimental values showed satisfactory correlation, indicating the validity and suitability of the models.

### 2.3. Analysis of Optimized Clozapine-Loaded Bilosomal Formulation

#### 2.3.1. Transmission Electron Microscopy (TEM) Investigation

**Figure 3:**
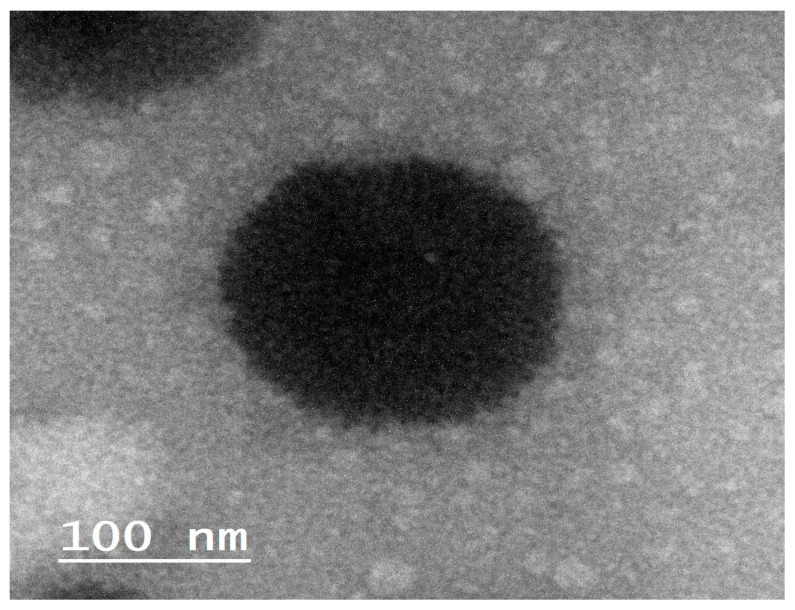
The TEM of the optimized formulation of bilosomes loaded with clozapine.

The TEM technique was employed to analyze the shape, size, and structure of the optimum clozapine bilosomes. The bilosomes were shaped as spherical vesicles with a distinct membrane, as demonstrated in Figure 3. In addition, TEM was employed to measure the particle size of the optimized bilosome formula. The size measured using the dynamic light scattering approach was found to be consistent with the value calculated by TEM.

#### 2.3.2. DSC Analysis

**Figure 4:**
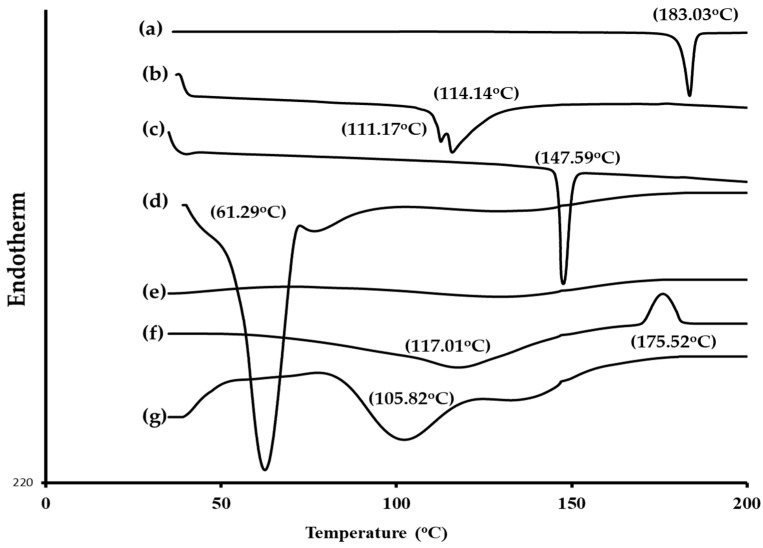
DSC thermograms of (a) pure clozapine, (b) PL, (c) cholesterol, (d) span 60, (e) clove oil, (f) SDC, and (g) optimized clozapine-loaded bilosomal formulation.

The DSC investigation was carried out in order to examine the possible interactions between the clozapine and excipients (Figure 4). A distinct endothermic peak at 183.03 °C was visible in the thermogram of the pure clozapine sample. This peak showed that clozapine is crystalline, and it showed its melting point. Figure 4 shows the thermal behavior of the PL, cholesterol, span 60, clove oil, and SDC, which were indicated by their different endothermic peaks. The optimized formulation clozapine thermogram did not show the expected endothermic peak reported in most thermograms of pure clozapine. These results suggested that the bilosomes had enhanced clozapine solubility, or that the clozapine molecules were dispersed uniformly across the bilosomes.

#### 2.3.3. In Vitro Release Study

**Figure 5:**
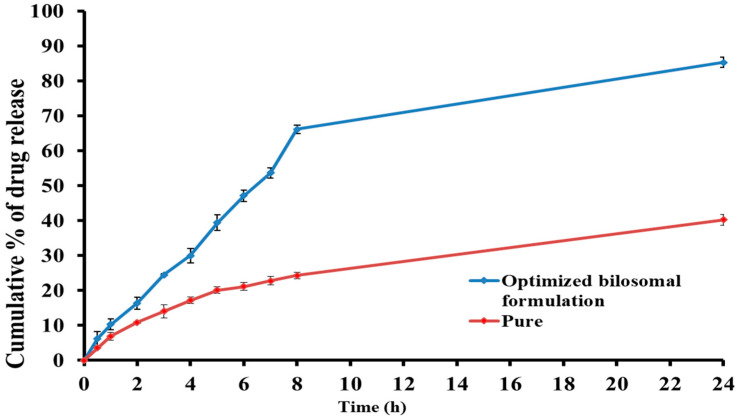
In vitro release of clozapine from the optimized clozapine-loaded bilosomal formulation compared to pure clozapine suspension.

Figure 5 illustrates the release of clozapine from the optimized bilosomal formulation in vitro. The pure clozapine demonstrated a notably decreased rate of drug release (*p* < 0.05) with about 40.19% within 24 h, which was attributed to the limited clozapine water solubility. In comparison, the optimized clozapine-loaded bilosomal formula released 85.27% of clozapine after 24 h. The sustained release of clozapine from the optimum bilosomal formulation was due to the high affinity of clozapine toward the hydrophobic counterpart of the bilosomal vesicles [14].

### 2.4. Development of Clozapine-Loaded Bilosomal In Situ Gel Formulations

In order to determine the impact that the mucoadhesive agent has on the properties of in situ gel formulations, a variety of mucoadhesive agents, such as carbopol 940 or HPMC, were combined with the P 407 gel base at a weight-to-weight ratio of 20%. The optimized bilosomes containing clozapine were included into the gel mixtures at various ratios of gel-to-liquid bilosomes (Table 3) in order to enhance the adherence of the formulation to the mucosal membrane. The selection of the optimized clozapine-loaded bilosomal in situ gel was based on the gel strength with an approved range of 25–50 s and the gelation time that would be close to 10 s at 34 °C [45]. The formulations (G2, G3, G5, and G6) that exhibited satisfactory gelation time and gel strength were chosen for further investigations.

### 2.5. Characterization of In Situ Gel Formulations Loaded with Clozapine Bilosomes

#### 2.5.1. pH Determination

Table 4 demonstrates the pH values for several in situ gel formulations. The pH values fell within the range of the normal pH levels of a nasal cavity. The pH of the gel formulations would promote non-irritant adherence to the nasal mucosa, leading to a prolonged effect with an intermediate permeability release rate [46].

#### 2.5.2. Measurement of Viscosity

Viscosity plays a critical role in optimizing intranasal formulations as it controls their flow properties, capacity to spread, drug release, and how long they stay on the nasal mucosa [47]. Table 4 displays the viscosity values that were obtained for different in situ formulations. The viscosity value of the in situ gel Formulation G6 was found to be 12,766.67 ± 230.94 cP, which was lower than that of the other in situ gel products investigated.

#### 2.5.3. Assessment of Spreadability

Spreadability is a quantitative measure of a gel’s ability to distribute and cover a certain surface area when applied to the nasal mucosa. It is a crucial component of intranasal formulation that permits consistent gel application and appropriate dosage administration of the medication [48]. The relationship between gel spreadability and the viscosity of different in situ gel preparations was found to be inversely proportional, as shown in Table 4. The G6 formulation demonstrated superior spreadability (3.89 ± 0.36 cm) and lower viscosity (12,766.67 ± 230.94 cP) in comparison to G2, G3, and G5.

#### 2.5.4. Investigation of Mucoadhesive Strength

The mucoadhesive strength values of G2, G3, G5, and G6 were determined, as reported in Table 4. Intranasal delivery may be considered suitable when the mucoadhesive strength values range from 4000–6000 dynes/cm^2^, according to the studies conducted by Wang et al. [49]. Wang et al. [49] suggested that having sufficient mucoadhesive strength might potentially enhance the duration of formulations staying in the nasal cavity, which could efficiently restrict their extraction and thus prolong the drug release. On the other hand, an excessively high degree of mucoadhesive strength might potentially harm the mucosal membrane.

The studied formulations displayed acceptable mucoadhesive strength values. Formulation G6 showed mucoadhesive strength values lower than other gel formulations, and this might be due its higher spreadability and lower viscosity.

#### 2.5.5. Study of In Vitro Release

**Figure 6:**
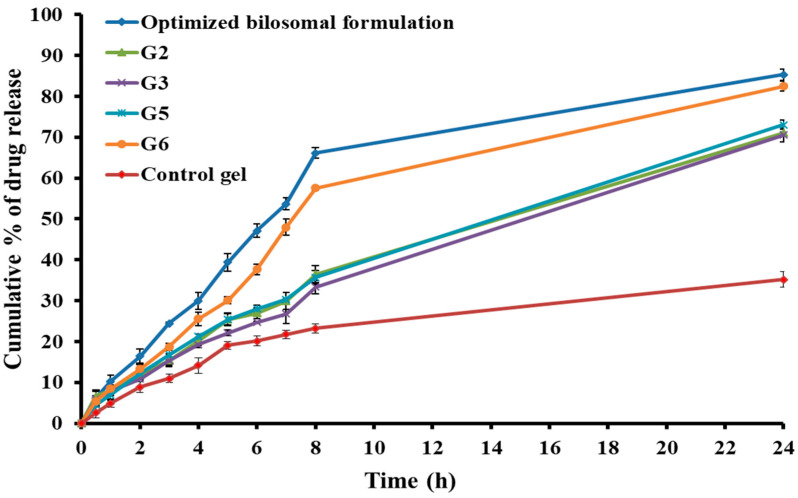
In vitro drug release from various clozapine-loaded bilosomal in situ gel formulations.

The clozapine release profile from the bilosomal in situ gel formulations (G2, G3, G5, and G6) compared to the optimized bilosome and pure clozapine-containing control gel is shown in Figure 6. It is possible that the increased viscosity of the bilosomal-based in situ gels, which resulted from the formation of a three-dimensional gel network, was the reason for their lower in vitro release rate of clozapine when compared to bilosomes [28]. Furthermore, the presence of bilosomal vesicles that encapsulate clozapine could explain why the in situ gel formulations showed a reduction in the rate of clozapine release. As the vesicles were encased in a polymeric membrane, the drug had to travel a longer distance before it could be released into the environment. Nevertheless, the incorporation of clozapine-loaded bilosomes into gel bases did not adversely affect the release of the medication. Indeed, more than 70% of the loaded clozapine was released from different in situ gel formulations during a 24 h period. Thus, considering the practical aspect, bilosomal in situ gels would be the preferable choice.

Upon evaluating the aforementioned criteria, it was found that Formulation G6 exhibited satisfactory characteristics. It also showed a pattern of medication release quite similar to the optimized liquid bilosomes. Formulation G6 was thus selected as the optimal clozapine-loaded bilosomal in situ gel and subjected to both in vivo and ex vivo permeation assessment.

### 2.6. Characterization of Optimized Bilosomal In Situ Gel Formulation Loaded with Clozapine

#### 2.6.1. Ex Vivo Permeation Study

**Figure 7:**
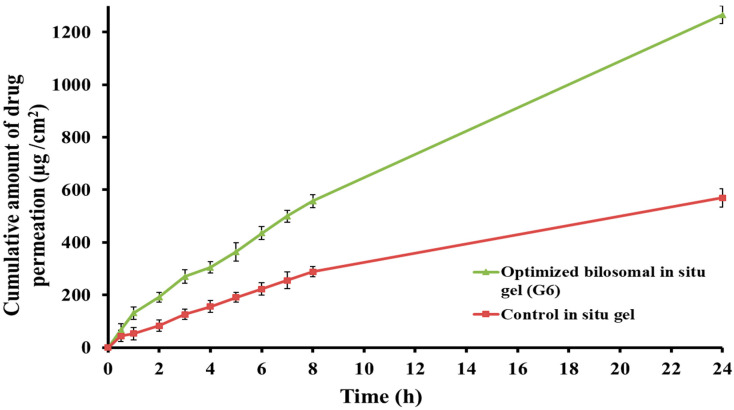
Ex vivo permeation of the clozapine from optimized bilosomal in situ gel Formulation G6 and control in situ gel.

The efficacy of clozapine in vivo was predicted by assessing its ex vivo permeability through the nasal mucosa from either the optimized bilosomal in situ gel (G6) or the control in situ gel. In comparison to the control in situ gel (which had the same gel composition as G6 but contained pure clozapine powder), Figure 7 illustrates how the addition of clozapine to the bilosomal in situ gel (G6) significantly increased the drug’s capacity to enter the nasal mucosa. There was a statistically significant difference (*p* < 0.001) in the observed outcome. The optimized G6 formulation demonstrated a drug penetration of 1265.78 ± 34.52 μg/cm^2^ through the nasal mucosa after 24 h in contrast to the 569.56 ± 33.83 μg/cm^2^ drug permeability observed in the control in situ gel that was loaded with clozapine.

The optimized bilosomal in situ gel formulation exhibited significantly higher J_ss_ and K_p_ values compared to the control in situ gel formulation, as shown in Table 5. This might potentially improve the optimized gel’s ability to significantly increase clozapine’s penetration performance and permeation across the nasal membrane (ER value of 2.21). The greater permeability of the optimized clozapine-loaded bilosomal in situ gel would be ascribed to the formulation’s components that compromised the lipid barrier of the nasal mucosa. Moreover, the proper viscosity of the preparation might influence the absorption of clozapine through the nasal mucosa. Furthermore, the presence of surfactant (span) and SDC facilitated the drug permeation by disrupting the tight junctions of the nasal membranes [30].

Teaima et al. pointed out that the penetration-enhancing action of essential oils such as clove oil will help to disrupt the lipid membranes and maximize the diffusion of drugs. In addition, the high vesicle deformability and elasticity as a result of using clove oil could facilitate the infiltration and easy squeezing of vesicles through the narrow openings of the biological membranes, hence enhancing their permeation [43]. The combined effects of surfactant (span), bile salt (SDC), and penetration enhancer (clove oil) would enable the bilosomal gel formulation to function as a drug reservoir, facilitating the effective transportation of clozapine across the nasal mucosa.

#### 2.6.2. Pharmacokinetic Study

**Figure 8:**
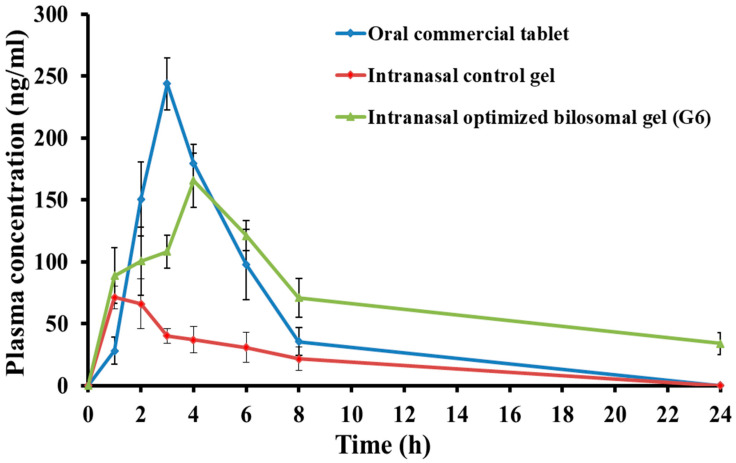
Plasma concentration–time curve of the clozapine.

Following the intranasal delivery of the optimized Formulation G6, the blood concentration–time profile of clozapine was captured, as depicted in Figure 8. This profile was compared to the intranasal control gel and the oral commercial tablet preparation. Our findings indicated that the optimized formulation exhibited significantly higher concentrations of clozapine in the plasma compared to the control gel. Table 6 reports how the maximum concentration (C_max_) of clozapine following administration of the optimal gel formulation via the nasal route, which was recorded at 165.49 ± 21.93 ng/mL, significantly exceeded the C_max_ observed with the control gel (71.30 ± 9.15 ng/mL). The bilosomal in situ gel formulation containing clozapine demonstrated a t_max_ value of 4 h, which significantly exceeded the t_max_ values of 1 h for the control gel and 3 h for the orally administered marketed tablets. The possibility of a controlled release mechanism through the bilosomal gel system was suggested by the observed increase in the t_max_ value of the optimized intranasal gel formulation (*p* < 0.05) in comparison to the other formulations under study.

In addition, the AUC_0–24_ of the optimized clozapine-loaded bilosomal gel formulation was 1739.46 ± 56.72 ng·mL^−1^·h. This value was much higher than the control in situ gel (354.00 ± 34.12 ng·mL^−1^·h) and the oral marketed formulation (936.21 ± 42.19 ng·mL^−1^·h) (*p* < 0.05). This suggests that the bilosomal gel systems, when applied intranasally, could lead to a longer release of the drug, resulting in a better level of bioavailability compared to the control gel and the orally administered tablets available in the market.

Moreover, the t_1/2_ of the optimized bilosomal gel formulation was dramatically enhanced compared to the oral marketed tablet and control gel. The t_1/2_ increased from 1.82 ± 0.32 h and 5.67 ± 0.48 h for the commercial tablet and control gel, respectively, to 9.74 ± 0.98 h for the optimal bilosomal gel formulation. The results demonstrated that the optimum bilosomal gel formulation exhibited a sustained release of clozapine and prolonged the presence of the drug in the systemic circulation, surpassing both the control gel and the oral marketed tablet.

The optimized bilosomal gel formulation effectively extended the MRT of the clozapine in the bloodstream. The MRT of the optimized formulation was 14.79 ± 0.88 h, while the MRT of the control gel and the oral market tablet were 7.42 ± 0.46 h and 4.45 ± 0.74 h, respectively. Remarkably, the inclusion of clozapine into the bilosomal gel led to a substantial increase in the systemic bioavailability of the clozapine. The optimized formulation exhibited a 4.9-fold enhancement in relative bioavailability compared to the control gel, and it was 1.9-fold greater than that of the orally marketed tablet. The results indicated that the systemic bioavailability of clozapine would be substantially enhanced when included into the bilosomal in situ gel compared to other studied preparations.

#### 2.6.3. Impact of Optimized Gel Formulation on the Levels of Hippocampal Dopamine, Serotonin, and BDNF

**Figure 9:**
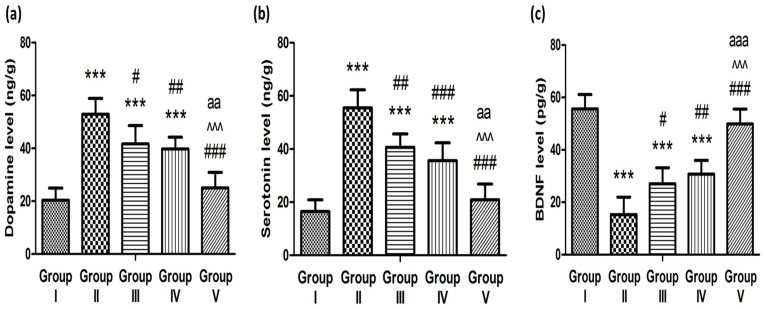
The effect of administration of the control in situ gel, oral marketed tablet, and optimized clozapine-loaded bilosomal in situ gel formulation on ketamine-induced dopamine (**a**), serotonin (**b**), and BDNF (**c**). *** *p* < 0.001 in comparison to Group I; ^#^ *p* < 0.05, ^##^ *p* < 0.01, and ^###^ *p* < 0.001 in comparison to Group II; ^^^^^ *p* < 0.001 in comparison to Group III; and ^aa^ *p* < 0.01 and ^aaa^ *p* < 0.001 in comparison to Group IV.

Figure 9a,b show that the hippocampal dopamine and serotonin levels were 2.6 and 3.4 times higher, respectively, in the rats induced with schizophrenia by ketamine than those in the control group. The significant elevation of dopamine and serotonin in the hippocampus after ketamine administration suggested that these neurotransmitters play a crucial role in the development of schizophrenia.

The administration of the oral market preparation (Group III), control in situ gel (Group IV), and optimized bilosomal in situ gel (Group V) significantly decreased the elevated dopamine levels by 1.26, 1.33, and 2.11 times, respectively, and it also decreased the elevated serotonin values by 1.36, 1.56, and 2.66 times, respectively, compared to the ketamine-induced schizophrenic animals. These results could support the affinity of clozapine to dopaminergic and serotonergic receptors in addition to the promoted neuroprotective properties of clozapine when embedded inside bilosomal in situ gels compared to other tested formulations. It has been reported that clozapine is the most effective anti-psychotic agent in restraining the neurotoxicity of NMDA antagonists [50].

Furthermore, BDNF has a crucial role in neuronal survival and synaptic plasticity [51]. Figure 9c demonstrates that the BDNF levels of schizophrenia-induced animals were decreased 3.6 fold in comparison to the control rats. This might be due to the role of ketamine in synaptic plasticity loss and the induction of schizophrenia [52]. Takahashi et al. and Durany et al. [53,54] reported that there was a remarkable reduction in the BDNF in the hippocampus of schizophrenic patients and that the BDNF expression in the hippocampus of schizophrenic patients would be elevated following treatment with anti-psychotic clozapine [54]. Our results showed that administration of the oral market preparation (Group III), control in situ gel (Group IV), and optimized bilosomal in situ gel (Group V) effectively increased the BDNF levels by 1.77, 2.01, and 3.26 times, respectively, compared to the ketamine-induced schizophrenic animals (Group II). Bai et al. pointed out that the up-regulation of BDNF expression after using atypical anti-psychotic clozapine could be related to the action of the drug as a serotonin receptor (2A) antagonist [55].

## 3. Materials and Methods

### 3.1. Materials

Clozapine was gifted from Delta Pharm Co., 10th of Ramadan, Egypt. Epikuron 200 phospholipid (PL) was obtained from Cargill Texturizing Solutions, Deutschland GmbH & Co., Hamburg, Germany. Cholesterol was purchased from BDH Chemicals Ltd., Poole, UK. Sodium deoxycholate (SDC) was supplied from Yunbang Pharm, Hunan, China. Clove oil was provided from Nefertari Natural Body Care, Cairo, Egypt. Methanol and chloroform were acquired from El-Nasr Pharmaceutical Chemicals, Cairo, Egypt. Hydroxypropyl methyl cellulose-K4M (HPMC) and carbopol 940 and were obtained from EIPICO, 10th of Ramadan, Egypt. Span 60, Span 80, and poloxamer 407 (P 407) were purchased from Sigma Chemical Co., St. Louis, MO, USA.

### 3.2. Preparation of Clozapine-Loaded Bilosomes

The preparation of bilosomal formulations loaded with clozapine was performed using a modified injection technique [56]. Briefly, in a separate vial, the PL, cholesterol (30 mg), span, clove oil, and clozapine were dissolved in a mixture of methanol and chloroform (1:2% *v*/*v*) as volatile organic solvents using a hot plate magnetic stirrer (MSH-20D, Daihan Scientific Co., Ltd., Seoul, Republic of Korea). SDC was dissolved in double-distilled water (5 mL) in another glass vial. At 70 °C, the solvent-containing solution was added dropwise on the SDC-containing aqueous solution, and the mixture was then kept stirred for 90 min or more till the organic solvents were evaporated. The formed bilosomal dispersions were cooled at the ambient temperature and then sonicated using an ice-cooled basket sonicator (USR 6, Julabo Labortechnik GmbH, Seelbach, Germany) within 4 min (30 s on and 30 s off). Thereafter, the bilosomes were stored in the refrigerator for further in vitro studies.

### 3.3. Definitive Screening Design (DSD)

Clozapine-loaded ultradeformable bilosomes were designed according to DSD using Design Expert^®^ software (version 11, Stat-Ease, Minneapolis, MN, USA) for studying the effect of various independent factors on bilosomal vesicle characteristics. Six factors including the PL amount (A), surfactant amount (B), SDC amount (C), clove oil amount (D), clozapine amount (E), and type of span (F) were selected as independent factors. The numerical factors were studied at their low, medium, and high levels, while the categorical factor (F) was studied at two levels (span 60 and span 80). Their effects on the studied dependent variables (particle size, entrapment efficiency, zeta potential, and cumulative release after 8 h) were examined using analysis of variance (ANOVA). It was recommended to reduce models and exclude insignificant models using the automatic selection algorithm to support hierarchy [57]. Fourteen experimental runs designed by DSD were performed in triplicate and their responses were examined, as listed in Table 7.

### 3.4. Characterization of Clozapine-Loaded, Lipid-Based Nano-Bilosomes

#### 3.4.1. Determination of the Particle Size and Zeta Potential

Each formulation was properly diluted with double-distilled water to assure the compatible intensity of the light scattering. The mean particle size and zeta potential were measured using a Zetasizer (Nano–ZS90, Malvern Instruments Ltd., Malvern, UK) at 25 °C. The experiment was applied three times, and the results were indicated as mean values ± standard deviation (S.D.).

#### 3.4.2. Determination of Entrapment Efficiency

The indirect process was followed to calculate the entrapment efficiency of clozapine, by which the free amount of clozapine was measured and subtracted from the theoretical value. Each clozapine-loaded bilosome (1 mL) was withdrawn and centrifuged at 18,000 rpm for 1 h at 4 °C using a cooling centrifuge (Z 326 K, Hermle Labortechnik GmbH, Wehingen, Germany). Then, the supernatant was separated, diluted with a corresponding medium as a blank (SDC-containing aqueous solution), and measured using a spectrophotometer (Genesys 10S UV-VIS, Thermo Spectronic, New York, NY, USA) at *λ*_max_ = 254 nm. The entrapment efficiency values were measured three times and calculated as follows [58,59]:

(5) $$Entrapment\;efficiency\;(\%)=\frac{Total\;clozapine\;amount-Free\;clozapine\;amount}{Total\;clozapine\;amount}\times 100.$$

#### 3.4.3. In Vitro Release Studies

The dialyzing technique was utilized to proceed the in vitro release of clozapine from the formulations under study. A shaking water bath (SW-20C, Julabo Labortechnik GmbH, Seelbach, Germany) was used after being adjusted at 100 rpm and 34 ± 0.5 °C. Briefly, the specific volume of clozapine-loaded bilosomes (containing clozapine equivalent to 10 mg) was added to a cellophane membrane dialysis tubing (molecular weight cut-off 12,000–14,000 Da), which was presoaked overnight in phosphate buffer saline (PBS) of a pH of 6.4. The dialysis tubes were placed in stoppered glass bottles including 100 mL of PBS with pH = 6.4. Successive samples (3 mL) were withdrawn at preset time intervals (0.5, 1, 2, 3, 4, 5, 6, 7, 8, and 24 h). An equal volume of a fresh release medium was returned to the receptor media to preserve the sink condition and keep the volume constant. The percentage of drug release was determined after spectrophotometric analysis at *λ*_max_ = 254 nm. The cumulative percentages of drug release against time were plotted.

### 3.5. Optimization of Clozapine-Loaded Bilosomes

By Design-Expert^®^ software, a numerical optimization technique was utilized to offer an optimum formula considering the significant factors and to obviate the non-significant ones. The optimizing criterion was to strengthen the entrapment efficiency, zeta potential, and cumulative release after 8 h % in addition to diminishing the particle size. The bilosomal formula was optimized according to its highest desirability value where a value close to one was selected. The affinity of the predicted and observed responses was examined. In addition, the optimized formulation was subjected to subsequent in vitro assay.

### 3.6. Characterization of Optimized Bilosomal Formulation

#### 3.6.1. Morphological Assessment Using Transmission Electron Microscope 

The morphological features of the optimized formula were inspected utilizing a transmission electron microscope (JEM-2100, JEOL, Tokyo, Japan). In brief, the bilosomal dispersion was properly diluted and then a single drop was withdrawn, which was then deposited on a carbon-coated copper grid and dried at room temperature. Subsequently, a droplet of 1% aqueous solution of phosphotungstic acid was used to cover the optimized bilosome that would be examined under microscope. The examination was done at 100 kV [60].

#### 3.6.2. Differential Scanning Calorimetry (DSC) 

The DSC profiles of the pure drug (clozapine), PL, cholesterol, span 60, clove oil, SDC, and optimized bilosomal formulation were studied using a DSC instrument (DSC-60, Shimadzu, Kyoto, Japan). This was done by placing small amounts of samples into aluminum pans. The tightly sealed pans were heated from 0 °C to 200 °C at 10 °C/min under a nitrogen flow (20 mL/min) [61].

### 3.7. Preliminary Study for Preparation of Intranasal Clozapine-Loaded Bilosomal In Situ Gels

Preparation of the in situ gels was carried out using the cold approach detailed by Choi et al. [62]. In summary, P 407 (20% *w*/*w*) was gently added to previously cooled distilled water at 4 °C with steady agitation for 15 min until translucent liquids were produced. At various concentrations, carbopol 940 (0.2%, 0.3%, and 0.4% *w*/*w*) or HPMC (1%, 1.5%, and 2% *w*/*w*) were progressively added to the P 407 polymeric solution (20% *w*/*w*) with agitation, and they were then cooled in an ice bath and six gel formulations were obtained. The optimized bilosomal formulation was then added to the produced in situ gels at various ratios of gel/liquid bilosome to investigate the gelation time and gel strength. The gel/liquid bilosome ratios were 2:1, 3:1, and 4:1 in the case of using carbopol 940 (0.2% and 0.3% *w*/*w*) and HPMC (1% and 1.5% *w*/*w*), as well as 1:1 and 2:1 in the case of using carbopol 940 (0.4% *w*/*w*) and HPMC (2% *w*/*w*). The optimized, in situ bilosomal gel formulation was chosen following pH, viscosity, spreadability, mucoadhesive strength, and in vitro clozapine release experiments.

### 3.8. Characterization of Clozapine-Loaded Bilosomal In Situ Gels

#### 3.8.1. Gelation Time Measurement

The gelation time was estimated using the gel inversion technique. Five grams of the prepared formulations were deposited in glass vials and transported to a heated water bath adjusted at 34 °C. The time to convert the created gels from a liquid state to a gel state was calculated.

#### 3.8.2. Gel Strength Determination

A 3.5 g weight was put on the top of each 5 g gel sample, and these were placed in a graduated measure to assess the gel strength. The period it took for the weight to go a distance of 3 cm inside the gel sample was recorded. The ideal time to record the gel strength was between 25 and 50 s [28].

#### 3.8.3. pH Measurement

The pH values were determined after dipping the glass electrode of a pH meter (5005, Jenco Instruments, San Diego, CA, USA) into each formulation at room temperature.

#### 3.8.4. Viscosity Evaluation

The in situ gels’ viscosity was determined by rotating the spindle of the viscometer (Visco Star-R, Fungilab S.A., Barcelona, Spain) at a speed of 10 rpm while it was submerged in the gels that were formed.

#### 3.8.5. Spreadability Assessment

The spreadability of the in situ gels was determined through the process of spreading 1 g of each sample in a circle, which had a 1 cm diameter, between two glass plates that were applied opposite to each other. The values of gel spreadability were determined after fixing a 0.5 kg weight on the plates [63].

#### 3.8.6. Mucoadhesive Strength Measurement

The adhesiveness ability of the formulations that were studied was assessed using fresh sheep nasal mucosa and pre-modified balance. The method measured the strength necessitated to remove the gel formulations from the nasal membrane. The balance’s right pan was substituted by a plastic cup. The balance pan placed in the left side was substituted by two glass vials whose bases were fixed opposite each other. Following the addition of two mucosal pieces that had been moistened with PBS (pH 6.4) to the base of each vial, the gel formulations that were being evaluated were sandwiched between the two mucosal components. It was necessary to apply a certain weight to the topmost vial for a period of two minutes in order to guarantee that the two glass vials were in close contact and the air trapped between the two vials was released. The plastic cup was progressively filled with water to separate the vials [64]. Mucoadhesive strength was calculated using the following formula:

(6) $$Mucoadhesive\;strength\;({dyne/cm}^{2})=\frac{g\times w}{A},$$

where g is the gravitational acceleration (980 cm/s^2^), w is the weight of the collected water (gram), and A is the nasal mucosa’s area (cm^2^).

#### 3.8.7. In Vitro Release Studies

In comparison to the control gel (which had the same gel composition of the optimized bilosomal gel but contained pure clozapine powder) and the optimized liquid bilosomal formulation, the in vitro release of clozapine from the in situ gels was carried out depending on the same procedures presented in Section 3.4.3.

The previous evaluation tests were implemented in triplicate and their values were expressed as the mean ± S.D. The formulation showing acceptable in vitro characterization was selected as the optimized bilosomal in situ gel formulation and was further evaluated in vivo.

### 3.9. Evaluation of Optimized Clozapine-Loaded Bilosomal In Situ Gel Formulation

#### 3.9.1. Ex Vivo Drug Permeation Study

A freshly extracted nasal tissue obtained from the nasal cavity of a sheep was utilized to investigate the ex vivo penetration features of clozapine. The fully cleansed mucosal membrane was fixed to a diffusion cell with a penetration area of 2.54 cm^2^. The donor compartment was filled with either the control gel or the optimized bilosomal in situ gel containing 10 mg of clozapine. The acceptor compartment comprised 100 mL of PBS (pH 6.4) and 0.02% sodium azide used as a preservative [65]. At different time durations, 1 mL of the samples was extracted from the acceptor compartment. Then, the samples were then accurately measured using spectrophotometry at a wavelength of 254 nm.

A graph was created by plotting the cumulative quantities of clozapine that permeated the per unit area (μg/cm^2^) against time. This graph was used to determine the slope of the linear part of the curve, which, in turn, helped determine the drug flux at a steady state (J_ss_). The permeability coefficient (K_p_) was obtained by dividing the flux by the initial amount of drug utilized. The enhancement ratio (ER) of the tested optimal formula compared to the controlled formula was determined by calculating the ratio of the J_ss_ of the formula to the J_ss_ of the controlled formula. The data are presented as the mean ± S.D. (n = 3).

#### 3.9.2. In Vivo Evaluation

##### Animals

This research comprised mature male albino rats weighing 250 g. The rats used in this investigation were acquired from the animal breeding facility at Zagazig University, which is situated in Egypt. The animals were kept in a controlled laboratory setting at a consistent room temperature and in a 12 h cycle of light and darkness. They were granted complimentary access to both nourishment and hydration. The rats had a minimum one-week period of acclimation before the tests began. The animals underwent care following the guidelines given by the Institutional Animal Care and Use Committee (IACUC) of the Faculty of Pharmacy at Zagazig University. This study proceeded following its approval (number ZU-IACUC/3/F/20/2023).

The optimized intranasal bilosomal in situ gel formulation, intranasal control in situ gel, and oral commercial tablet (Clozapex^®^, Apex Pharma, New Cairo, Egypt) were adjusted as was previously calculated by [66]. The animals were randomly allocated into five groups, where each group contained six animals (n = 6).

Group I consisted of rats that were used as a control group and were provided with a regular diet at the appropriate periods.

Group II consisted of rats that were induced with schizophrenia by the injection of ketamine.

Group III consisted of animals with schizophrenia that were administered by the oral marketed tablets.

Group IV consisted of animals with schizophrenia that were administered by the intranasal control in situ gel.

Group V consisted of animals with schizophrenia that were administered by the optimized intranasal bilosomal in situ gel formulation.

##### Inducing Schizophrenia in Rats

Ketamine functions as an N-methyl-D-aspartate (NMDA) receptor antagonist and is frequently employed to elicit schizophrenia-like symptoms in animal models through extended intraperitoneal administration. Sub-anesthetic dosages of ketamine can induce the psychomimetic effects in rats. As a result, schizophrenia was induced in rats by ketamine injection into their abdominal cavity every day for 14 days. Each injection had a dose of 30 mg/kg [67]. Group II animals were administered a daily dose of ketamine alone at a concentration of 30 mg/kg for a duration of 14 days. Group III, IV, and V animals were administered a daily dose of ketamine alone (30 mg/kg) for the initial 7 days. Subsequently, they received a daily dose of both ketamine (30 mg/kg) and the studied formulations for the next 7 days, with a 30 min interval between the two administered treatments.

##### Pharmacokinetic Parameters Calculation

The subsequent calculation of the pharmacokinetic parameters was conducted following the administration of the tested formulations. On the final day of the in vivo study, blood samples were obtained from certain animal groups (III, IV, and V) from the lateral tail vein. Heparinized tubes were used to collect the samples at different time intervals at 0, 1, 2, 3, 4, 6, 8, and 24 h. The blood samples were centrifuged for 10 min at a speed of 3000 rpm, which led to the separation of clear plasma. The plasma samples were kept at −20 °C until analyzed. The separated samples were thawed at room temperature then subjected to high-performance liquid chromatography (HPLC) analysis (Waters 2690 Alliance HPLC system equipped with a Waters 996 photodiode array detector, Milford, MA, USA). Afterward, each sample (400 μL) was mixed with acetonitrile (1 mL) then vortexed using a vortex mixer (Purimix, Cryste-Novapro, Bucheon, Republic of Korea) for 2 min. Thereafter, the samples were centrifuged at 15,000 rpm for a duration of 10 min at a temperature of 4 °C using a cooling centrifuge. The supernatants were exposed to evaporation by means of a stream of nitrogen gas. The samples were reconstituted by adding the mobile phase (120 μL) and then filtered using micro syringe filters. The experiment employed a mobile phase consisting of a solution containing 0.3% triethyl amine and 0.05 M monobasic potassium phosphate (pH = 3.5) mixed with acetonitrile (65:35% *v*/*v*). The experiment maintained a persistent flow rate of the mobile phase (1 mL/min). Samples (100 μL) were injected into a C18 thermal column with dimensions of 4.6 × 250 mm and a particle size of 5 μm while keeping the temperature constant at the surrounding environment. The investigation utilized a photodiode array detector (996, Waters, Milford, MA, USA) with a detection wavelength of 280 nm. Degassing of the mobile phase was done by sonication.

The parameters of pharmacokinetic study were assessed using the non-compartmental approach with the aid of the PKsolver tool, an add-in for Microsoft Excel, version 2. The profiles of plasma concentration against time were used to measure the maximum plasma concentration (C_max_) and the time necessitated to reach this concentration (t_max_). The calculation of the apparent elimination rate constant (K_el_) required examining the final slope exhibited in the plasma concentration–time curves. The trapezoidal approach was used to determine both the area under the plasma concentration–time curve (AUC _0–24 h_) and the area under the curve from time zero to infinity (AUC _0–∞_). The calculations were performed to calculate the elimination half-life (t_1/2_), mean residence time (MRT), and relative bioavailability. The pharmacokinetic results were reported as the mean ± S.D. (n = 6).

##### Analysis of the Enzyme-Linked Immunosorbent Assay (ELISA)

The rats were euthanized, after which their hippocampal tissues were precisely removed and immediately washed in a solution of cooled saline. The levels of dopamine, serotonin, and brain-derived neurotrophic factor (BDNF) in the hippocampus of different treated animal groups were determined using commercially available ELISA kits. The specific kits used were MBS7214676 (MyBiosource, Inc., San Diego, CA, USA) for dopamine, MBS725497 (MyBiosource, Inc., San Diego, CA, USA) for serotonin, and MBS355345 (MyBiosource, Inc., San Diego, CA, USA) for BDNF. The ELISA tests were performed in exact accordance with the manufacturer’s instructions.

### 3.10. Analysis of Data Using Statistical Methods

The parameters underwent statistical analysis using one way ANOVA, followed by Tukey’s post hoc test. The analysis was conducted using GraphPad Prism^®^ software (San Diego, CA, USA), version 5.

## 4. Conclusions

This study signified the validity of DSD in successfully developing an optimized bilosomal formulation that has considerable characteristics. The DSD results showed the significant impact of utilizing a high PL amount on increasing the particle size and entrapment efficiency of bilosomal vesicles. Using high amounts of surfactant (span), bile salt (SDC), and penetration enhancer (clove oil) facilitated decreasing the particle size and increasing the entrapment efficiency of bilosomes in addition to increasing the release of clozapine. Moreover, bilosomes containing span 60 displayed a higher encapsulation and release of clozapine than those containing span 80. On the other side, incorporation of optimized liquid bilosome into mucoadhesive in situ gel bases showed the feasibility of HPMC (2% *w*/*w*)/P 407 (20% *w*/*w*) mixture in producing satisfactory viscosity, spreadability, and mucoadhesive strength than a carbopol 940/P 407 mixture. In comparison to the control gel, the combined effects of span 60, SDC, clove oil, and HPMC enabled the optimized G6 to function as a superior drug reservoir, facilitating the transportation of clozapine through the nasal mucosa. Pharmacokinetic and biochemical studies on ketamine-induced schizophrenic rats confirmed the prolonged presence of clozapine in systemic circulation and the effective anti-psychotic action of clozapine in restraining the neurotoxicity of ketamine when embedded in bilosomal gels and applied intranasally.

## Figures and Tables

**Table 1:** ANOVA analysis of the studied dependent responses.

Particle Size						
Source	Sum of Squares	df	Mean Square	F-Value	*p*-Value	
Model	90,468.22	5	18,093.64	20.65	0.0002	Significant
A-PL amount	28,291.76	1	28,291.76	32.29	0.0005	
B-Surfactant amount	8219.69	1	8219.69	9.38	0.0155	
C-SDC amount	32,341.97	1	32,341.97	36.91	0.0003	
D-Clove oil amount	7182.40	1	7182.40	8.20	0.0211	
E-Clozapine amount	14,432.40	1	14,432.40	16.47	0.0036	
Residual	7010.48	8	876.31			
Cor total	97,478.70	13				
**Entrapment Efficiency %**						
**Source**	**Sum of Squares**	**df**	**Mean Square**	**F-Value**	***p*-Value**	
Model	7187.38	6	1197.90	100.39	<0.0001	Significant
A-PL amount	2069.39	1	2069.39	173.43	<0.0001	
B-Surfactant amount	1682.83	1	1682.83	141.03	<0.0001	
C-SDC amount	1198.41	1	1198.41	100.44	<0.0001	
D-Clove oil amount	1841.14	1	1841.14	154.30	<0.0001	
E-Clozapine amount	461.16	1	461.16	38.65	0.0004	
F-Type of span	188.26	1	188.26	15.78	0.0054	
Residual	83.53	7	11.93			
Cor total	7270.91	13				
**Zeta Potential**						
**Source**	**Sum of Squares**	**df**	**Mean Square**	**F-Value**	***p*-Value**	
Model	776.70	2	388.35	33.06	<0.0001	Significant
B-Surfactant amount	239.12	1	239.12	20.36	0.0009	
E-Clozapine amount	537.58	1	537.58	45.77	<0.0001	
Residual	129.20	11	11.75			
Cor total	905.90	13				
**Cumulative Release After 8 h**						
**Source**	**Sum of Squares**	**df**	**Mean Square**	**F-Value**	***p*-Value**	
Model	2456.58	5	491.32	21.25	0.0002	Significant
A-PL amount	934.21	1	934.21	40.40	0.0002	
B-Surfactant amount	358.83	1	358.83	15.52	0.0043	
C-SDC amount	571.08	1	571.08	24.70	0.0011	
D-Clove oil amount	149.23	1	149.23	6.45	0.0347	
F-Type of span	264.62	1	264.62	11.44	0.0096	
Residual	184.99	8	23.12			
Cor total	2641.57	13				

df, degree of freedom; PL, phospholipid; and SDC, sodium deoxycholate.

**Table 2:** The predicted and experimental values of the optimized clozapine-loaded bilosomal formulation.

Factors	Optimized Level	
A-PL amount (mg)	104.69	
B-Surfactant amount (mg)	100	
C-SDC amount (mg)	45.33	
D-Clove oil amount (mg)	97.76	
E- Clozapine amount (mg/mL)	5	
F-Type of span	Span 60	
**Responses**	**Predicted**	**Experimental**
Particle size (nm)	164.33	179.60 ± 12.72
Entrapment efficiency (%)	88.57	86.91 ± 1.36
Zeta potential (mV)	47.34	48.60 ± 1.04
Cumulative drug release after 8 h	69.65	66.13 ± 1.26

PL, phospholipid; SDC, sodium deoxycholate.

**Table 3:** Composition and assessment of the various clozapine-loaded bilosomal in situ gel formulations according to gelation time and gel strength.

Formulation	Gel Base Type	Mucoadhesive Agent	Gel:Bilosome Ratio (*w*/*w*)	Gelation Time (sec)	Gel Strength (sec)
G1	P 407 (20% *w*/*w*)	Carbopol 940 (0.2% *w*/*w*)	2:1	No gelation	-
			3:1	No gelation	-
			4:1	No gelation	-
G2	P 407 (20% *w*/*w*)	Carbopol 940 (0.3% *w*/*w*)	2:1	No gelation	-
			3:1	No gelation	-
			4:1	9.89 ± 0.72	35.19 ± 1.30
G3	P 407 (20% *w*/*w*)	Carbopol 940 (0.4% *w*/*w*)	1:1	No gelation	-
			2:1	6.12 ± 1.13	47.13 ± 1.45
G4	P 407 (20% *w*/*w*)	HPMC (1% *w*/*w*)	2:1	No gelation	-
			3:1	No gelation	-
			4:1	No gelation	-
G5	P 407 (20% *w*/*w*)	HPMC (1.5% *w*/*w*)	2:1	No gelation	-
			3:1	No gelation	-
			4:1	11.27 ± 2.03	33.69 ± 1.27
G6	P 407 (20% *w*/*w*)	HPMC (2% *w*/*w*)	1:1	No gelation	-
			2:1	13.28 ± 1.97	27.72 ± 1.51

**Table 4:** Properties of various clozapine bilosomal in situ gel preparations.

Formulation	Gel Base Type	Mucoadhesive Agent	Gel:Formula Ratio	pH	Viscosity (cP)	Spreadability (cm)	Mucoadhesive Strength (dyne/cm^2^)
G2	P 407 (20% *w*/*w*)	Carbopol 940 (0.3% *w*/*w*)	4:1	6.25 ± 0.36	15,400.00 ± 264.58	2.90 ± 0.72	4727.16 ± 63.77
G3	P 407 (20% *w*/*w*)	Carbopol 940 (0.4% *w*/*w*)	2:1	6.17 ± 0.14	17,566.67 ± 115.47	2.76 ± 0.15	5132.68 ± 39. 62
G5	P 407 (20% *w*/*w*)	HPMC (1.5% *w*/*w*)	4:1	6.58 ± 0.60	14,733.33 ± 152.75	3.28 ± 0.12	4407.81 ± 43.65
G6	P 407 (20% *w*/*w*)	HPMC (2% *w*/*w*)	2:1	6.60 ± 0.27	12,766.67 ± 230.94	3.89 ± 0.36	4273.93 ± 30.17

**Table 5:** Ex vivo permeation studies of clozapine from the control in situ gel and optimized bilosomal in situ gel Formulation G6.

Parameters	Optimized Bilosomal In Situ Gel (G6)	Control In Situ Gel (Contain Pure Drug)
J_ss_ (μg/cm^2·^h^−1^)	50.86 ± 1.28	23.03 ± 1.81
K_p_ × 10^−3^ (cm/h)	9.95 ± 0.83	4.51 ± 0.24
ER	2.21	-

J_ss_, drug flux at steady state; K_p_, permeability coefficient; and ER, enhancement ratio.

**Table 6:** Pharmacokinetic parameters of the clozapine.

Parameters	Oral Commercial Tablet	Intranasal Control Gel	Intranasal Optimized Bilosomal Gel (G6)
C_max_ (ng/mL)	243.61 ± 20.92	71.30 ± 9.15	165.49 ± 21.93
t_max_ (h)	3	1	4
K_el_ (h^−1^)	0.38 ± 0.09	0.12 ± 0.11	0.07 ± 0.01
t_1/2_ (h)	1.82 ± 0.32	5.67 ± 0.48	9.74 ± 0.98
AUC_0–24 h_ (ng·mL^−1^·h)	936.21 ± 42.19	354.00 ± 34.12	1739.46 ± 56.72
AUC_0-∞_ (ng·mL^−1^·h)	1029.51 ± 49.92	530.30 ± 40.49	2215.74 ± 63.80
MRT (h)	4.45 ± 0.74	7.42 ± 0.46	14.79 ± 0.88
Relative bioavailability (%)	264.46	-	491.37

**Table 7:** The bilosomal formulations given by DSD and experimental data responses.

Run	A: PL Amount (mg)	B: Surfactant Amount (mg)	C: SDC Amount (mg)	D: Clove Oil Amount (mg)	E: Clozapine Amount (mg/mL)	F: Type of Span	Particle Size (nm)	Entrapment Efficiency (%)	Zeta Potential (mV)	Cumulative Release After 8 h (%)
1	250	55	30	50	10	Span 80	335.40 ± 27.15	55.29 ± 0.19	32.38 ± 0.45	34.06 ± 2.15
2	100	55	50	0	5	Span 80	238.70 ± 9.82	41.93 ± 1.71	41.71 ± 1.37	53.67 ± 1.30
3	100	10	10	100	10	Span 80	382.30 ± 18.10	30.77 ± 1.92	25.69 ± 1.92	33.44 ± 1.81
4	100	100	30	100	5	Span 60	215.50 ± 23.06	78.67 ± 1.04	49.96 ± 0.98	67.91 ± 2.04
5	400	55	10	100	15	Span 60	455.00 ± 21.67	70.21 ± 1.75	30.52 ± 1.18	30.41 ± 1.69
6	250	100	50	100	15	Span 80	285.20 ± 17.72	81.46 ± 0.62	30.54 ± 1.92	46.56 ± 1.65
7	400	100	50	0	10	Span 60	345.00 ± 25.44	88.57 ± 1.19	40.86 ± 1.33	41.97 ± 0.38
8	250	10	10	0	5	Span 60	407.70 ± 20.17	26.56 ± 1.86	33.54 ± 1.57	24.28 ± 2.15
9	100	100	10	0	15	Span 60	405.10 ± 15.93	24.99 ± 0.81	30.75 ± 0.79	47.97 ± 0.30
10	250	55	30	50	10	Span 60	295.60 ± 17.92	65.27 ± 1.57	40.55 ± 2.11	51.64 ± 0.81
11	400	10	50	100	5	Span 80	325.50 ± 10.85	83.59 ± 1.52	41.95 ± 1.05	34.73 ± 2.14
12	400	10	30	0	15	Span 80	535.00 ± 22.35	32.64 ± 2.01	21.87 ± 1.95	15.15 ± 1.29
13	100	10	50	50	15	Span 60	292.50 ± 9.15	39.08 ± 0.63	25.76 ± 1.31	51.01 ± 1.65
14	400	100	10	50	5	Span 80	405.50 ± 12.37	78.74 ± 1.25	45.60 ± 1.05	24.30 ± 1.88

PL, phospholipid; SDC, sodium deoxycholate. The zeta potential values have negative signs. The values in the table are expressed as absolute values.

## Data Availability

The data presented in this study are contained within the article.

## Supplementary Materials

The following supporting information can be downloaded at: https://www.mdpi.com/article/10.3390/ph17101404/s1, Figure S1. DSD plots of the influence of (a) the PL amount on particle size, (b) the surfactant amount on particle size, (c) the SDC amount on particle size, (d) the clove oil amount on particle size, and (e) the clozapine amount on particle size. Figure S2. DSD plots of the influence of (a) the PL amount on entrapment efficiency, (b) the surfactant amount on entrapment efficiency, (c) the SDC amount on entrapment efficiency, (d) the clove oil amount on entrapment efficiency, (e) the clozapine amount on entrapment efficiency, and (f) the type of span on entrapment efficiency. Figure S3. DSD plots of the influence of (a) the span amount on zeta potential and (b) the clozapine amount on zeta potential. Figure S4. DSD plots of the influence of (a) the PL amount on drug release percent after 8 h, (b) the surfactant amount on drug release percent after 8 h, (c) the SDC amount on drug release percent after 8 h, (d) the clove oil amount on drug release percent after 8 h, and (e) the span type on drug release percent after 8 h.
