# Supplementary material for: Evaluation of Mucoadhesive Nano-Bilosomal In Situ Gels Containing Anti-Psychotic Clozapine for Treatment of Schizophrenia: In Vitro and In Vivo Studies
Source: Pharmaceuticals (Basel). 2024 Oct 21;17(10):1404. doi: 10.3390/ph17101404 (PMC11510079; doi:10.3390/ph17101404)
Supplement: Supplementary file 1 [file pharmaceuticals-17-01404-s001.zip › pharmaceuticals-3258599-supplementary.pdf]

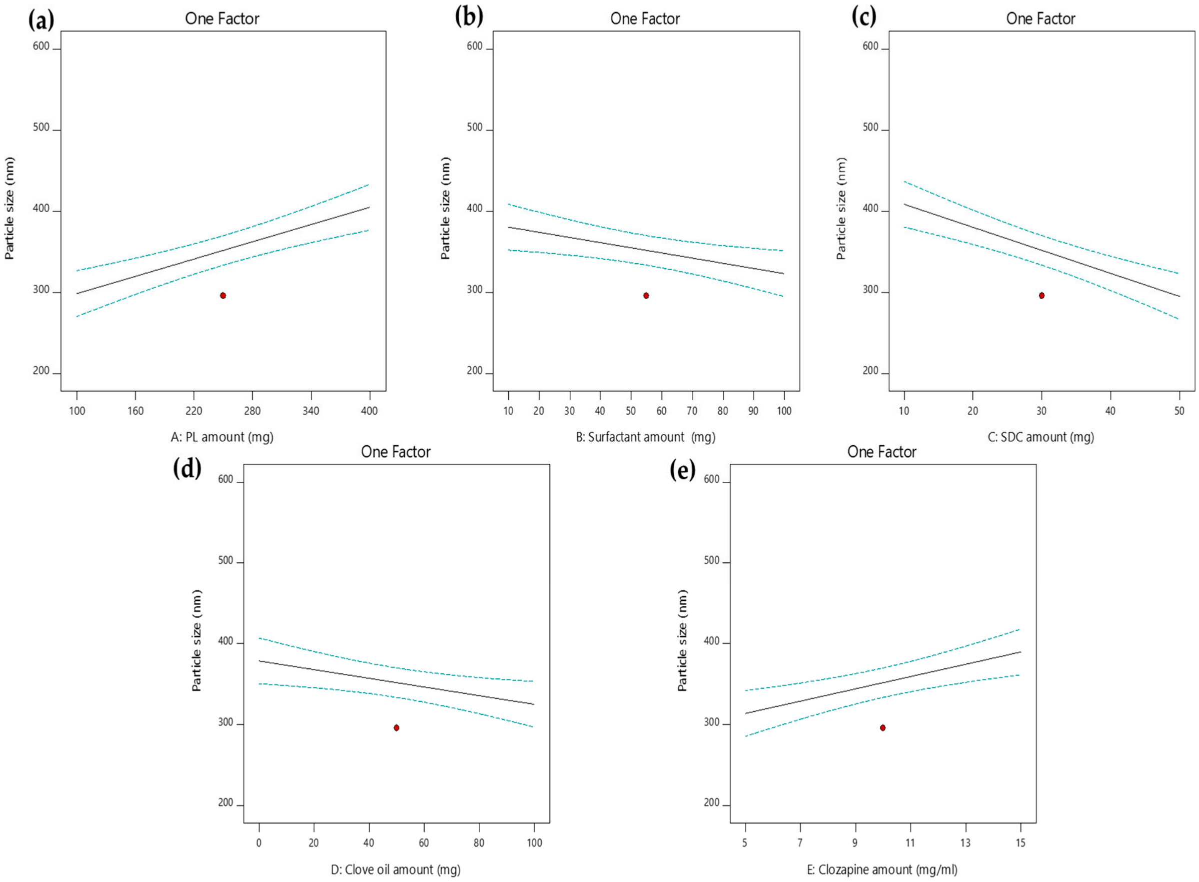

Figure S1. DSD plots showing the influence of (a) PL amount on particle size (b) surfactant amount on particle size (c) SDC amount on particle size (d) clove oil amount on particle size (e) clozapine amount on particle size.

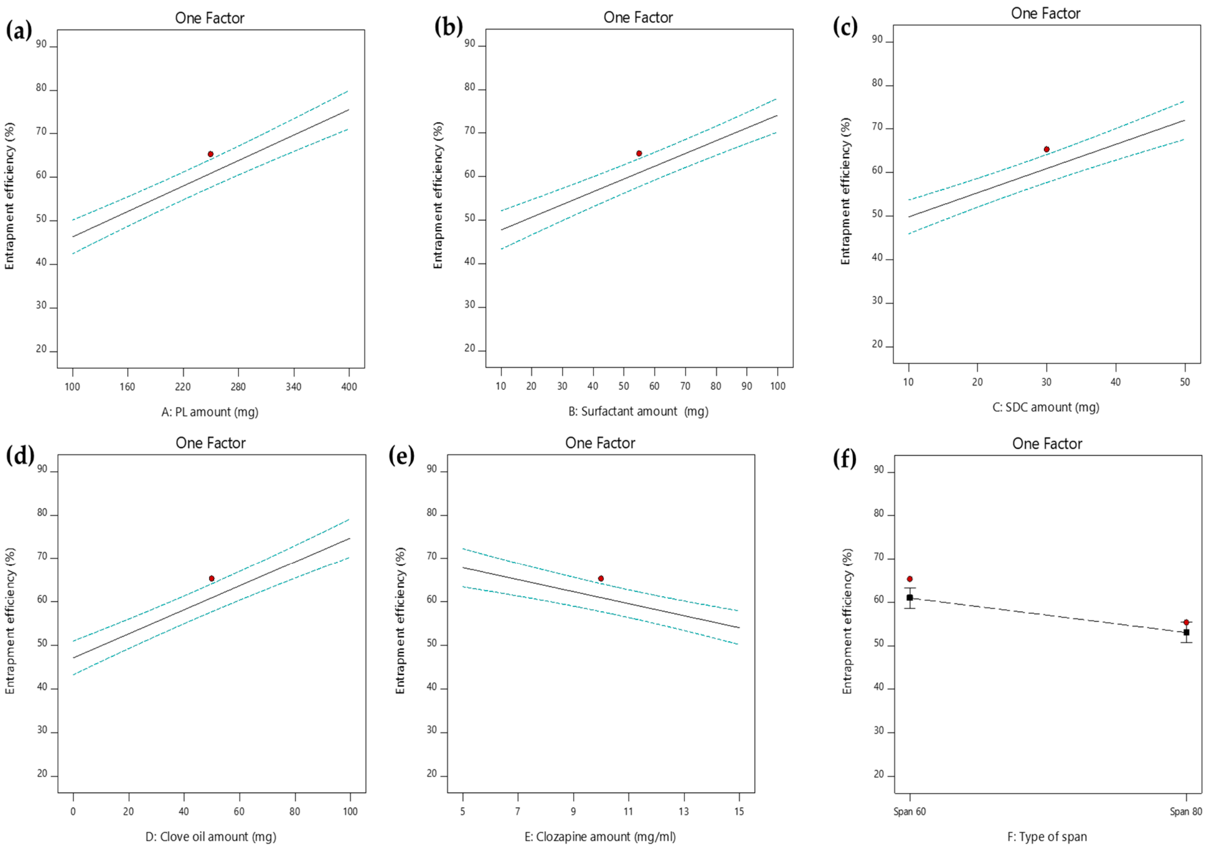

Figure S2. DSD plots showing the influence of (a) PL amount on entrapment efficiency (b) surfactant amount on entrapment efficiency (c) SDC amount on entrapment efficiency (d) clove oil amount on entrapment efficiency (e) clozapine amount on entrapment efficiency (f) type of span on entrapment efficiency.

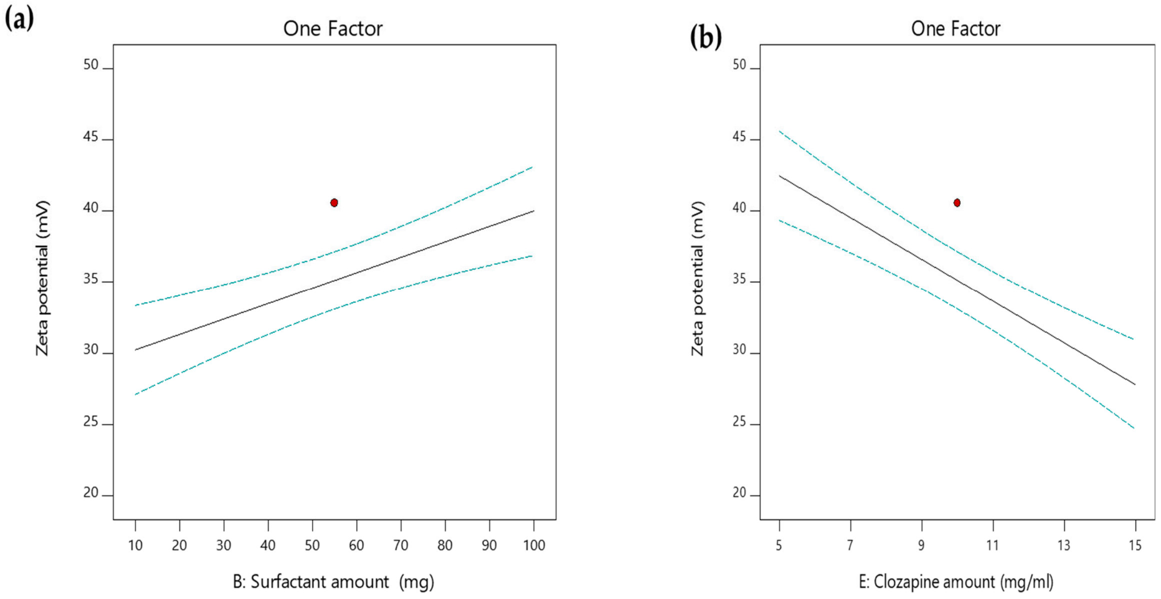

Figure S3. DSD plots showing the influence of (a) span amount on zeta potential (b) clozapine amount on zeta potential.

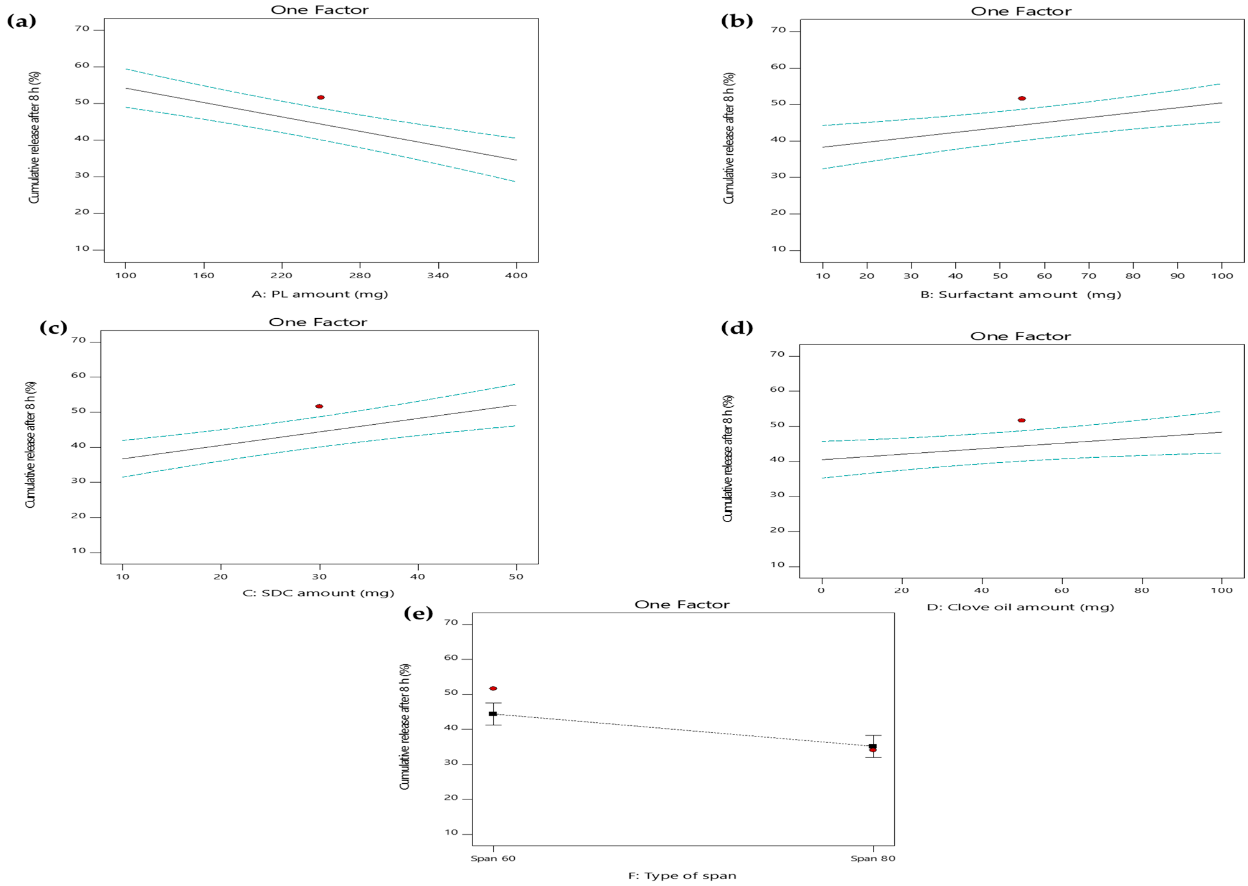

Figure S4. DSD plots showing the influence of (a) PL amount on drug release percent after 8 h (b) surfactant amount on drug release percent after 8 h (c) SDC amount on drug release percent after 8 h (d) clove oil amount on drug release percent after 8 h (e) span type on drug release percent after 8 h.
